# Targeting PHB1 to inhibit castration-resistant prostate cancer progression in vitro and in vivo

**DOI:** 10.1186/s13046-023-02695-0

**Published:** 2023-05-20

**Authors:** Junmei Liu, Ranran Zhang, Tong Su, Qianqian Zhou, Lin Gao, Zongyue He, Xin Wang, Jian Zhao, Yuanxin Xing, Feifei Sun, Wenjie Cai, Xinpei Wang, Jingying Han, Ruixi Qin, Laurent Désaubry, Bo Han, Weiwen Chen

**Affiliations:** 1 Department of Biochemistry and Molecular Biology, School of Basic Medical Sciences, Cheeloo College of Medicine, Shandong University, Jinan, China; 2 The Key Laboratory of Experimental Teratology, Ministry of Education and Department of Pathology, School of Basic Medical Sciences, Cheeloo College of Medicine, Shandong University, Jinan, China; 3 Department of Thoracic Surgery, Qilu Hospital of Shandong University, Jinan, China; 4 Research Center of Basic Medicine, Jinan Central Hospital, Shandong First Medical University, Jinan, China; 5 School of Basic Medical Sciences, Cheeloo College of Medicine, Shandong University, Jinan, China; 6 Department of Pathology, Qilu Hospital of Shandong University, Jinan, China; 7 INSERM, UMR 1260, Regenerative Nanomedicine, University of Strasbourg, FMTS (Fédération de Médecine Translationnelle de L’Université de Strasbourg), Strasbourg, France

**Keywords:** CRPC, Prohibitin, FL3, Enzalutamide

## Abstract

**Background:**

Castration-resistant prostate cancer (CRPC) is currently the main challenge for prostate cancer (PCa) treatment, and there is an urgent need to find novel therapeutic targets and drugs. Prohibitin (PHB1) is a multifunctional chaperone/scaffold protein that is upregulated in various cancers and plays a pro-cancer role. FL3 is a synthetic flavagline drug that inhibits cancer cell proliferation by targeting PHB1. However, the biological functions of PHB1 in CRPC and the effect of FL3 on CRPC cells remain to be explored.

**Methods:**

Several public datasets were used to analyze the association between the expression level of PHB1 and PCa progression as well as outcome in PCa patients. The expression of PHB1 in human PCa specimens and PCa cell lines was examined by immunohistochemistry (IHC), qRT-PCR, and Western blot. The biological roles of PHB1 in castration resistance and underlying mechanisms were investigated by gain/loss-of-function analyses. Next, in vitro and in vivo experiments were conducted to investigate the anti-cancer effects of FL3 on CRPC cells as well as the underlying mechanisms.

**Results:**

PHB1 expression was significantly upregulated in CRPC and was associated with poor prognosis. PHB1 promoted castration resistance of PCa cells under androgen deprivation condition. PHB1 is an androgen receptor (AR) suppressive gene, and androgen deprivation promoted the PHB1 expression and its nucleus-cytoplasmic translocation. FL3, alone or combined with the second-generation anti-androgen Enzalutamide (ENZ), suppressed CRPC cells especially ENZ-sensitive CRPC cells both in *vitro* and in vivo. Mechanically, we demonstrated that FL3 promoted trafficking of PHB1 from plasma membrane and mitochondria to nucleus, which in turn inhibited AR signaling as well as MAPK signaling, yet promoted apoptosis in CRPC cells.

**Conclusion:**

Our data indicated that PHB1 is aberrantly upregulated in CRPC and is involved in castration resistance, as well as providing a novel rational approach for treating ENZ-sensitive CRPC.

**Supplementary Information:**

The online version contains supplementary material available at 10.1186/s13046-023-02695-0.

## Introduction

Prostate cancer (PCa) is a malignant tumor that poses a severe threat to males [1]. The aberrant activation of AR signaling plays a vital role in PCa progression and androgen deprivation therapy (ADT) is the primary therapy for locally advanced or metastatic PCa, but most patients will gradually develop from androgen-dependent prostate cancer (ADPC) into castration-resistant prostate cancer (CRPC) after initial treatment [2]. The occurrence and development of CRPC is a complex process involved and driven by multiple molecular pathways [3], in which reactivation of the AR signal pathway is a critical driver of CRPC. Although AR-targeted drugs such as Enzalutamide (ENZ) as well as drugs targeting other CRPC-driver genes, such as AKT inhibitor ipatasertib, PARP inhibitors olaparib and rucaparib, have improved the clinical outcome of CRPC patients, disease resistance remains a serious challenge [3–6,7]. Therefore, searching for novel targets and targeted drugs, is still an urgent problem to be solved.

Prohibitin (PHB1/PHB) is a ubiquitously expressed and evolutionarily conserved protein. PHB1 gene is located on chromosome 17q21 and encodes a 32 kDa protein composed of 272 amino acids [8]. PHB1 gene was initially cloned based on the ability of its own 3’UTR to induce growth arrest in mammalian cells and was found to be unrelated to the function of the protein itself [9]. Multiple studies have found that PHB1 is a multifunctional molecular chaperone/scaffold protein involving in cell growth, proliferation, apoptosis, and metabolism. PHB1 protein is localized primarily to mitochondria, plasma membrane and nucleus. Of note, the subcellular localization of PHB1 determines its differential effects [10, 11]. Mitochondrial PHB1 is involved in maintaining mitochondrial homeostasis and suppressing apoptosis [12–14]; nuclear PHB1 functions as a cofactor to mediate transcriptional regulation through interacting with several transcription factors, either directly or indirectly [15–19]; cell membrane-located PHB1 is required for K-Ras-mediated C-Raf activation [20, 21]. In recent years, a large number of studies have shown that PHB1 expression is upregulated in a variety of human malignancies, including lung cancer, diffuse large B-cell lymphomas (DLBCL), cervical cancer, bladder cancer, glioblastoma, endometrial cancer, and breast cancer, etc. [22–27]. For example, PHB1 is significantly increased in plasma membrane of paclitaxel-resistant subline A549TR compared to A549 cells and mediates paclitaxel resistance via over-activating C-Raf [28]. PHB1 is overexpressed in human bladder cancer tissues and mainly localized to mitochondria. The overexpression of PHB1 was significantly associated with poor prognosis of bladder cancer patients [25]. In human breast cancer cell lines, MCF-7 and T47D, PHB1 is mainly localized in the nucleus, but camptothecin treatment promotes its translocation from the nucleus to the mitochondria in response to apoptosis signal [29]. In summary, the roles of PHB1 in human malignancies seem to be closely related to the cell types and its subcellular distribution.

Due to its low mutation rate and essential function, PHB1 is a suitable drug target [30, 31]. Flavaglines are a class of natural products isolated from the medicinal plant *Aglaia*, which exerts a unique anti-cancer activity by binding to PHB1/PHB2 [32]. FL3, a synthetic derivative of flavaglines with higher tumor cell specificity and lower normal cell cytotoxicity compared to natural products, has presented potent tumor suppressive effect by targeting PHB1 in many cancers such as DLBCL, urothelial carcinoma of bladder (UCB), colorectal cancer (CRC), and glioblastoma cancer, etc. [23, 33–36]*.* By contrast, the role of PHB1 in PCa is still controversial. Bevan et al*.* characterized PHB1 as a novel corepressor of AR on prostate tumor growth and an androgen suppressive target gene. Overexpression of PHB1 inhibits androgen-stimulated growth of LNCaP cells both in vitro and in vivo [19, 37, 38]. Contrarily, Zhu et al*.* reported that mitochondria-located PHB1 suppresses TGF-β induced apoptosis in CRPC cell line PC-3 [39]. In addition, Ummanni and Cho, respectively, reported that PHB1 was upregulated in PCa specimens and was positively correlated with the degree of malignancy [40, 41]. Therefore, we speculate that PHB1 may be involved in the castration resistance. In this study, we explored the role of PHB1 in CRPC and assessed the effect of FL3 to treat CRPC.

## Materials and methods

### Reagents

Fetal bovine serum (FBS), charcoal-stripped fetal bovine serum (CSS, depleted androgen and any other steroid), Bovine pituitary extract (BPE), and cell culture mediums were purchased from Gibco (NY, USA). ENZ was purchased from MedChemExpress (NJ, USA). FL3 was synthesized in Dr. Laurent Désaubry’s lab according to a described procedure [42]. DHT was purchased from Meilunbio (Dalian, China). Epidermal growth factor (EGF) was obtained from Peprotech (NJ, USA). Glutamine PenStrep was obtained from Invitrogen (CA, USA). Serial dilutions of all drugs were made using DMSO.

### Bioinformatics analysis

The mRNA expression data of PHB1 for differential expression analysis was extracted from GEO (http://www.ncbi.nlm.nih.gov/geo) database and cBioPortal (http://www.cbioportal.org/) database. The data used for Kaplan–Meier survival analysis were from cBioPortal database and TCGA (https://www.cancer.gov/about-nci/organization/ccg/research/structural-genomics/tcga/using-tcga) database. Data plotting and statistical analysis were performed using GraphPad Prism 8.0 (GraphPad Software, USA). T-test was used to compare the PHB1 mRNA expression from database between the different groups. For Kaplan–Meier curves, p-value was generated by Log-rank tests.

### Immunohistochemistry(IHC)

The cancer-adjacent normal tissues and PCa specimens were collected from patients undergoing radical prostatectomy at Qilu Hospital, Shandong University (Jinan, China) and then were made into tissue microarray (TMA). TMA slides staining with H&E were reviewed by two pathologists according to the WHO histologic classification of PCa. After that, the IHC staining was performed with General-purpose two-step immunohistochemical detection kit (PV-9000, Zhongshan Golden Bridge Biotechnology Co, Beijing, China). For PHB1 expression in tissues, the staining intensity was scored from 1 to 3: 1, weak; 2, moderate; 3, strong. The information of antibody used is summarized in Supplementary Table S2.

### Cell lines and cell culture

Human prostate epithelial cell line RWPE-1 was purchased from the National Collection of Authenticated Cell Cultures (Shanghai, China). PCa cell lines LNCaP, VCaP, DU145, C4-2B, 22Rv1, and PC-3 were purchased from American Type Culture Collection (ATCC; Manassas, VA, USA). RWPE-1 cells were cultured in Keratinocyte serum-free medium supplemented with EGF, BPE, and 1% glutamine PenStrep. LNCaP, C4-2B, and 22Rv1 cells were cultured in RPMI-1640 medium with 10% FBS or CSS. PC-3 cell was cultured in F12-K medium with 10% FBS or CSS. VCaP and DU145 cells were cultured in DMEM medium with 10% FBS. LNCaP-AI is the androgen-independent subline of LNCaP, which was derived from passaging LNCaP cells in RPMI-1640 medium supplemented with 10% CSS for at least 3 months [43]. All cells were cultured at 37 °C in an atmosphere of 5% CO_2_ and used up to 15 passages maximum. After the last experiment, all cells were confirmed mycoplasma free using GMyc-PCR Mycoplasma Test Kit (40601ES10, YeSen Biotech, Shanghai, China).

### qRT-PCR

Total RNA was isolated using TRIzol reagent (Invitrogen, CA, USA), and 1 μg of total RNA was used as the template for the first strand cDNA synthesis using the ReverTra Ace qPCR RT Kit (TOYOBO, Osaka, Japan). qPCR was conducted with a FastStart Universal SYBR Green Master Mix (Roche, Mannheim, Germany). The data was normalized to β-actin in each sample. The primer sequences used are listed in Supplementary Table S1.

### Western blot

Whole cell lysates were prepared by lysing the cells in ice-cold RIPA buffer (P0013C, Beyotime, Shanghai, China) with 1% protease inhibitor cocktail (NCM Biotech, Suzhou, China). 20 μg total protein was separated by SDS-PAGE and transferred to the PVDF membrane, which were then incubated with corresponding antibodies. Information on antibodies is summarized in Supplementary Table S2.

### Plasmids, siRNA, and cell transfection

Expression vector pENTER-PHB1 and its control vector were constructed by Vigene Bioscience (Shandong, China). siNC and siPHB1 were synthesized by Jikai company (Shanghai, China). The siRNA sequences are listed in Supplementary Table S3. Lipofectamine 3000 (Invitrogen, CA, USA) was used for transient transfection following the manufacturer’s instruction. The transfection efficiency was confirmed by qRT-PCR and Western blot.

### MTS assay

Cell viability and cell proliferation were determined by MTS assay with MTS Cell Proliferation Assay Kit (BestBio, Shanghai, China). LNCaP cells (3.0 × 10^3^ cells/well), C4-2B cells (2.0 × 10^3^ cells/well), 22Rv1 (1.5 × 10^3^ cells/well), or PC-3 cells (1.5 × 10^3^ cells/well) were plated in 96-well plate. After cells adhered to the wall, 10 μl MTS was added to each well at 0, 24, 48, and 72 h, respectively, and incubated at 37 °C for 2 h. Then the absorbance was measured at 490 nm. Cell viability was expressed as the percentage of absorbance of control. Experiments were performed in triplicate and repeated three times.

### Transwell assay

Transwell invasion/migration assays were performed using Transwell Chambers with or without coated Matrigel (Corning, NY, USA). RPMI-1640 or F12-K medium with 10% FBS or 10% CSS was added to the lower chamber as a chemoattractant for cells. LNCaP (10.0 × 10^4^ cells/well), C4-2B (10.0 × 10^4^ cells/well), 22Rv1 (7.0 × 10^4^ cells/well), or PC-3 cells (7.0 × 10^4^ cells/well) were plated in the upper chamber with RPMI-1640 or F12-K medium. After 24 h of incubation, the invasive or migrated cells were fixed with 4% paraformaldehyde (Biosharp, Beijing, China) and stained with crystal violet solution (Solarbio, Beijing, China). 5 fields of view were randomly selected for counting transmembrane cells under the light microscope using a 40 × magnification. Experiments were repeated independently three times.

### Plate colony formation assay

The clonogenic ability of PCa cells was measured by plate colony formation assay. LNCaP (300 cells/well), C4-2B (300 cells/well), 22Rv1 (200 cells/well) or PC-3 cells (200 cells/well) were plated in 6-well plate. 14 d after initial seeding, cells were fixed with 4% paraformaldehyde and stained with crystal violet solution. Colonies containing more than 50 cells were counted and plotted. Experiments were repeated independently three times.

### Subcellular fractionation

Nucleus and cytosol proteins were separated using Nuclear and Cytoplasmic Protein Extraction Kit (Beyotime Biotechnology, Nantong, China) following the manufacturer’s instruction. plasma membrane extracts were prepared using Minute™ Plasma Membrane Protein Isolation and Cell Fractionation Kit (Invent Biotechnologies, Eden Prairie, USA) according to the manufacturer’s manual.

### Immunofluorescence (IF)

IF was performed as previously described [44]. Pre-treated LNCaP (4 × 10^4^ cells/well) and C4-2B (2 × 10^4^ cells/well) cells were seeded on glass coverslips in 24-well plates, respectively. Mitochondrial labeling was performed using MitoTracker Deep Red FM (40743ES, Yeasen Biotech, Shanghai, China) following the manufacturer’s instruction. Nucleus was stained with DAPI (H-1200, VECTASHIELD, CA, USA). Antibodies used were summarized in Supplementary Table S2. Cells were observed under Laser Confocal Microscope LSM 980 (Zeiss, Oberkochen, Germany). Images taken were appropriately processed with the ZEN software program. Experiments were repeated three independent times.

### Co-Immunoprecipitation (Co-IP)

Co-IP assays were performed according to the instruction of BeyoMag™ Protein A/G Magnetic Beads for IP (Beyotime, Shanghai, China). 10 μg antibody in a 1:50 dilution was incubated with 20 μl Protein A/G Magnetic Beads for 1 h at temperature. The cell lysate or nuclear extract, leaving 50 µl as input, was incubated with antibody-coated immunomagnetic beads at 4 ℃ overnight. The immuno-complex was collected and analyzed by Western blot. The information on antibodies is summarized in Supplementary Table S2. Independent, sequential experiments were repeated three times.

### Chromatin Immunoprecipitation (ChIP)

ChIP assays were performed using Magna ChIP A/G (No Controls) kit (17–10,085, Sigma-Aldrich, Darmstadt, Germany) according to the instruction of manufacturer. Briefly, cells were cultured in 10 cm dishes and cross-linked with 1% formaldehyde for 10 min. Then, 10 × glycine buffer was added to the cell suspension at room temperature for 10 min to quench the fixation reaction. After washing the cells with PBS, cell pellets were resuspended in lysis buffer from the kit with Protease Inhibitor Cocktail II. Lysed cells were sonicated at 750 W, 25% power (10 s on, 10 s off) for 3 runs, then set lysed cells on ice for 2 min, up to a total of 8 runs. Immunoprecipitation was performed by adding 5 µg of antibody to the lysate. The associated DNA fragments were purified and used as the templates for qPCR. The used primer sequences and antibody information are listed in Supplementary Table S1 and S2, respectively. Experiments were repeated three independent times.

### Tumor xenografts

5-week-old male BALB/c nude mice were purchased from Weitonglihua Biotechnology (Beijing, China). A total of 6.0 × 10^6^ C4-2B cells were mixed with Matrigel (1:1) and injected subcutaneously into the mice (*n* = 6/group). After being surgically castrated, mice were randomized into 4 groups (*n* = 6/group) and treated with indicated drugs in indicated concentrations. Drugs were administered once every two days via oral gavage or intraperitoneally. Body weight and tumor volume were measured and recorded twice a week. Tumor tissues were harvested and weighed after 15 times of treatment. Tumor volume was calculated with the following formula: tumor volume = length × width^2^ × 0.5. All animal experiments followed a protocol approved by the Shandong University Animal Care Committee (Document No. ECSBMSSDU2019-2–019).

### Flow cytometry

Dead Cell Apoptosis Kit (Invitrogen, CA, USA) was used according to the manufacturer's instruction. Cells after treatment were harvested and were dealt with Annexin V-FITC and propidium iodide, followed by apoptosis analysis with CytoFLEX S (1720610S-2, BECKMAN COULTER, CA, USA). Independent experiments were repeated three times.

### Pattern drawing

The pattern diagram was drawn using the Figdraw software (ResearchHome, Zhejiang, China).

### Statistical analysis

In the in vitro experiment, experiments were carried out at least in triplicate to confirm reproducibility and presented as mean ± SD. In the xenograft study, tumor sizes were served as the primary response measure when the mice were sacrificed. Statistical analysis was carried out with GraphPad Prism 8.0 using a t-test and two-way ANOVA. Significance was determined at *P < 0.05, **P < 0.01 and ***P < 0.001.

## Results

### PHB1 expression is upregulated in CRPC and is associated with poor prognosis.

**Fig. 1:**
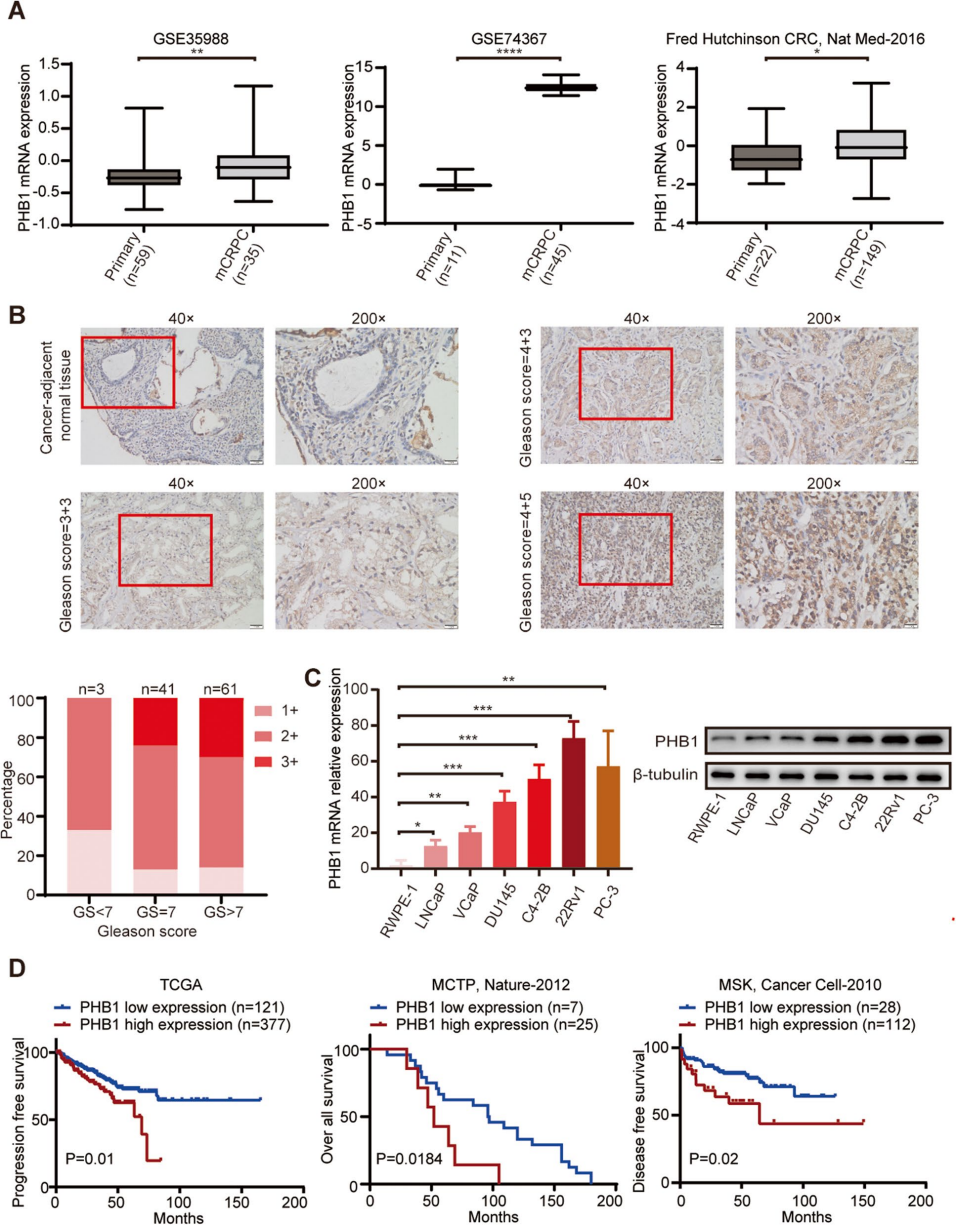
The expression of PHB1 is elevated in CRPC and is associated with poor prognosis. **A** Expression of PHB1 in CRPC tissues compared with primary PCa samples in GSE35988 (left), GSE74367 (middle), and Fred Hutchinson CRC, Nat Med-2016 (right) public datasets. *P < 0.05, **P < 0.01, ****P < 0.0001. **B** Representative IHC images of PHB1 in PCa tissues (above) and the quantification of IHC scores (below). Magnified images from the regions marked by rectangles were shown on the right. **C** The mRNA and protein levels of PHB1 in RWPE-1, LNCaP, VCaP, C4-2B, 22Rv1, and PC-3 cells were determined by qRT-PCR (left) and Western blot (right). Western blot was performed with the indicated antibodies. For qRT-PCR, β-actin was used as the reference gene. For Western blot, β-tubulin was used as a loading control. *P < 0.05, **P < 0.01, **P < 0.001. **D** Kaplan–Meier survival analysis of PCa cases from TCGA and cBioPortal PCa cohort. Left: the progression free survival analysis of the TCGA PCa cohort (*p* = 0.01; Log-rank test); Middle: the overall survival analysis of MCTP, Nature-2012 dataset from cBioPortal database (*p* = 0.0184; Log-rank test); Right: the disease-free survival analysis of MSK, Cancer Cell-2010 dataset from cBioPortal database (*p* = 0.02; Log-rank test)

To explore its role in castration resistance, we first conducted differential expression analysis using public datasets to investigate the association between the mRNA expression level of PHB1 and PCa progression. As shown in Fig. S1A-C, PHB1 expression was upregulated in localized PCa tissues compared with benign prostate tissues (GSE35988) [45]. Higher PHB1 level was associated with higher Gleason score (*P* = 0.0054) (GSE46602) [46]. While the analysis of the TCGA PCa cohort (*n* = 498) showed no significant difference in the PHB1 levels between groups with Gleason score < 7 and that with Gleason score ≥ 7, the PHB1 level was correlated with pathological T staging and clinical T staging (Pathological T staging: T2 vs. T3, *P* < 0.01; Clinical T staging: T1 vs. T3, *P* < 0.01, T1 vs. T4, *P* < 0.05). More importantly, analysis of three independent datasets (GSE35988, GES74367 and Fred Hutchinson CRC, Nat Med-2016) [45, 47, 48] showed significantly increased PHB1 expression in CRPC tissues compared to primary PCa tissues (Fig. 1A). Further, IHC examination on 105 clinical PCa specimens and 5 cancer-adjacent normal tissues showed that PHB1 expression was significantly higher in PCa tissues than that in adjacent normal tissues and was positively correlated with the Gleason score (Fig. 1B). We also examined the basal expression of PHB1 in 6 PCa cell lines. Compared with the benign prostate epithelial cell line RWPE-1, the expression of PHB1 was significantly upregulated in the 4 CRPC cell lines, DU-145, C4 -2B, 22Rv1 and PC-3, yet slightly upregulated in the ADPC cell line LNCaP and the androgen-sensitive PCa cell line VCaP with wild-type AR [49, 50] (Fig. 1C). Finally, we performed Kaplan–Meier analysis to assess prognosis using the RNAseq data from TCGA database and cBioPortal database, and the Log-rank test was used to compare differences between high PHB1 expression (PHB1^**hi**^) group and low PHB1 expression (PHB^**lo**^) group. As shown in Fig. 1D, compared with PHB^**lo**^ group, the PHB1^**hi**^ group had shorter progression free survival (TCGA), overall survival (MCTP, Nature-2012) [45], and disease-free survival (MSK, Cancer Cell-2010) [51]. The above results suggested that increased PHB1 expression positively correlates with the grades of PCa and may participate in the castration resistance.

### PHB1 is involved in the development of CRPC

**Fig. 2:**
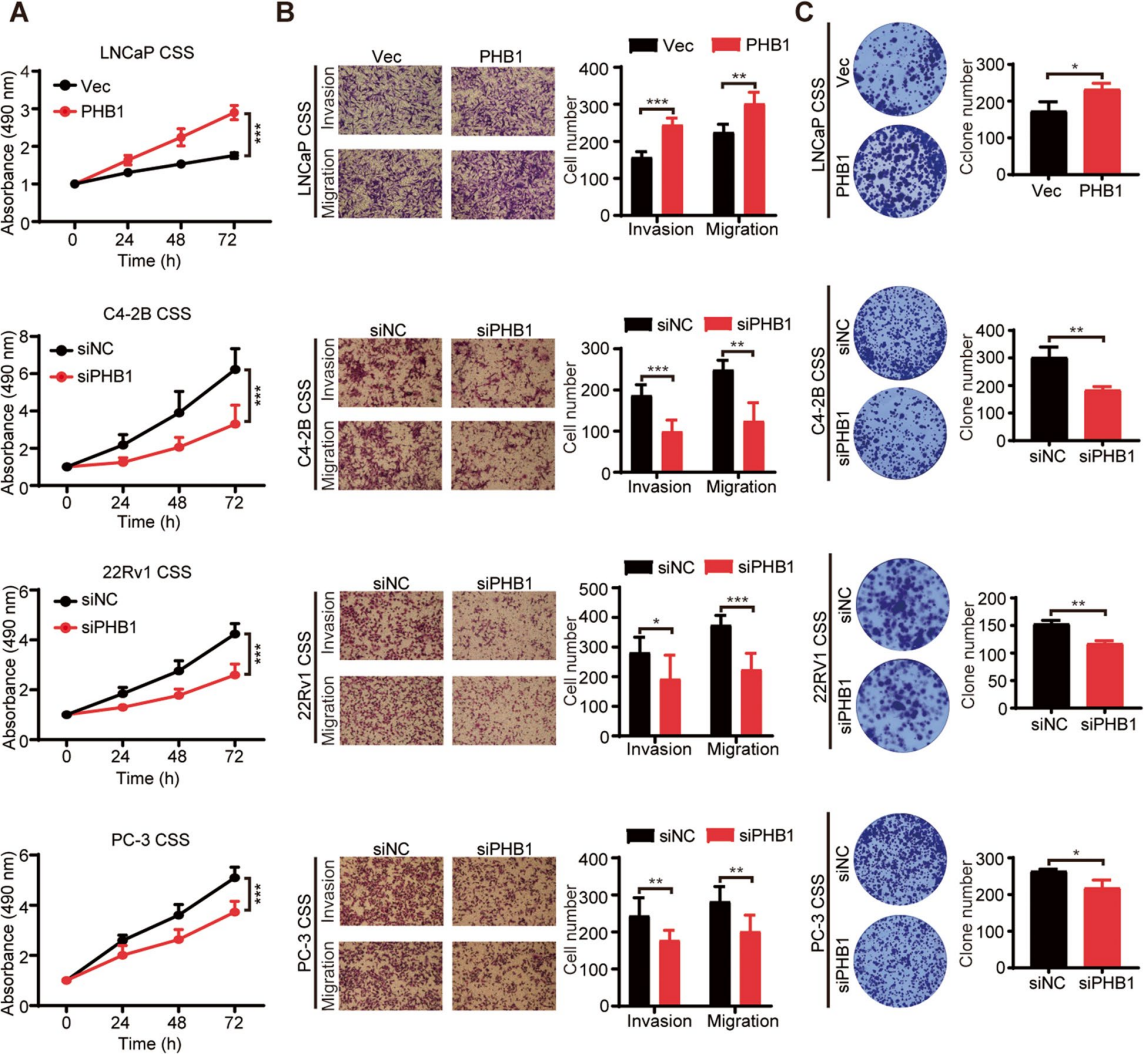
PHB1 promotes castration resistance. After CSS culture for 3 d, LNCaP cells were transfected with pENTER/pENTER-PHB1, C4-2B, 22Rv1, and PC-3 cells were transfected with siNC/siPHB1, respectively. **A** Cell proliferation was determined by MTS assay. The absorbance was read at 490 nm at the indicated time points and normalized to 0 h values. ***P < 0.001. **B** Invasion and migration capacity of PCa cells were determined by transwell assay. Left panel: representative images; Right panel: quantitative results of triplicate experiments. *P < 0.05, **P < 0.01, ***P < 0.001. **C** Cell clonogenic ability was determined by plate colony formation assay. Left panel: representative images; Right panel: quantitative results of triplicate experiments. *P < 0.05, **P < 0.01. *CSS* Charcoal-stripped serum. *h* Hours

To explore whether PHB1 is involved in the castration resistance, we selected ADPC cell line LNCaP with lower PHB1 level and 3 CRPC cell lines with more PHB1 expression, C4-2B, 22Rv1 and PC-3, to perform a series of gain/loss-of-function experiments. All the cells were firstly grown in the medium with 10% CSS for 3d to mimic the ADT environment, and then PHB1 was overexpressed in LNCaP but downregulated in C4-2B, 22Rv1, and PC-3 cells, respectively. MTS, transwell, and plate colony formation assays were carried out respectively to determine the changes in cell function. As shown in Fig. S2A and Fig. 2, under CSS condition, PHB1 overexpression significantly enhanced the proliferation, invasion, migration, and clonogenic ability of LNCaP cells, whereas PHB1 knockdown obviously inhibited the cell growth, invasion, migration, and clonogenic growth of C4-2B, 22Rv1, and PC-3 cells, respectively. In addition, we also repeated the gain/loss-of-function analysis under FBS condition. As shown in Fig. S2B-D, all the results showed similar trends as under CSS condition, and to a lesser extent under FBS condition. Together, these data strongly suggested that PHB1 is involved in the development of CRPC.

### Androgen depletion upregulates the PHB1 expression and promotes its nucleus-cytoplasmic translocation

**Fig. 3:**
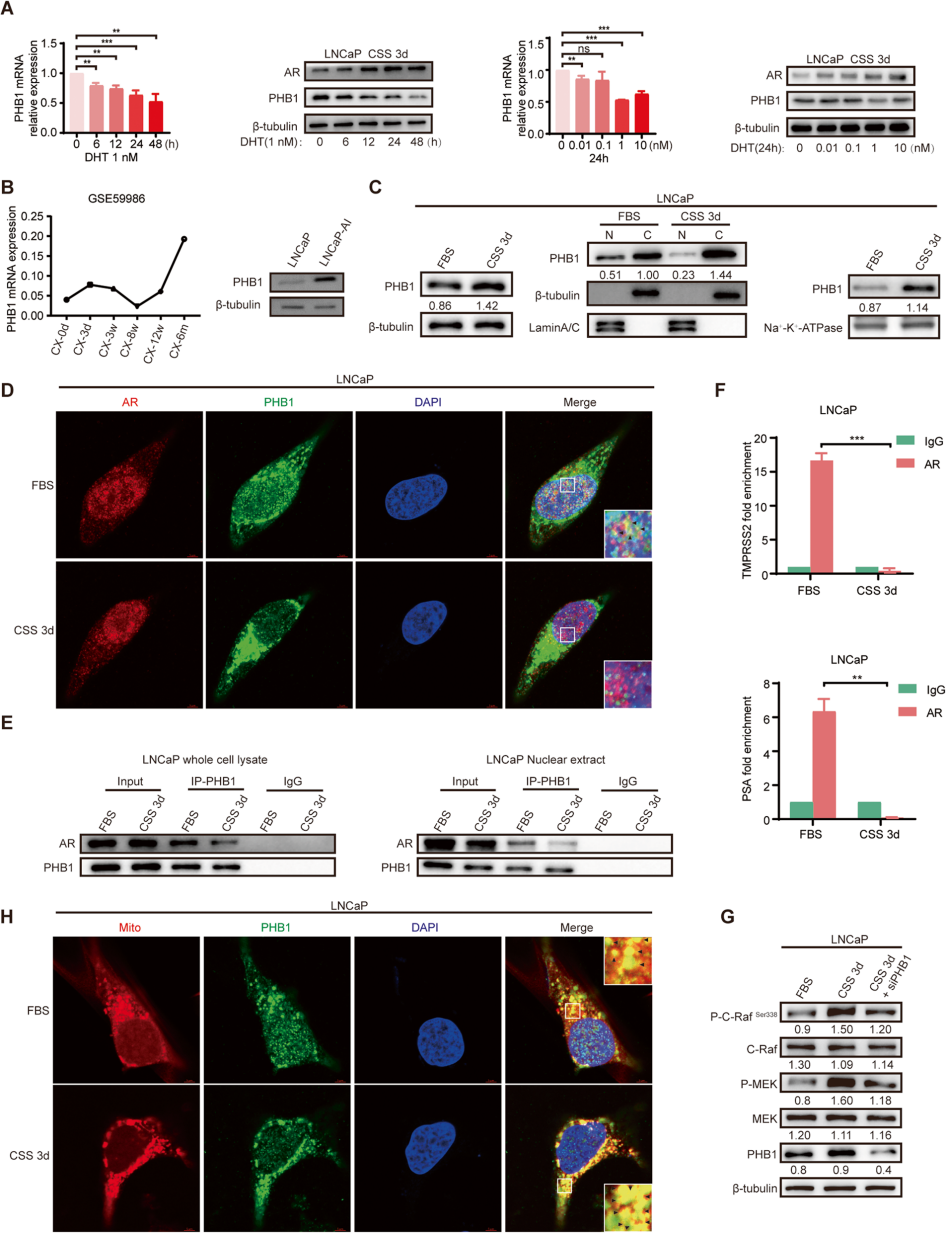
The androgen deprivation upregulates PHB1 expression and alters its subcellular distribution. **A**. The mRNA and protein levels of PHB1 in LNCaP cells were determined by qRT-PCR and Western blot. LNCaP cells were treated with 1 nM DHT at the indicated time points (left two panels) or treated with 0.01, 0.1, 1, and 10 nM DHT for 24 h (right two panels). Western blot was performed with the indicated antibodies. For qRT-PCR, β-actin was used as the reference gene. For Western blot, β-tubulin was used as a loading control. **P < 0.01, ***P < 0.001. d, days. h, hours. CSS, charcoal-stripped serum. **B** The mRNA and protein levels of PHB1 in LNCaP cells following chronic androgen deprivation were analyzed by public dataset and Western blot. Left: mRNA levels of PHB1 in public dataset (GSE59986); Right: protein levels of PHB1 in LNCaP and LNCaP-AI cells. Western blot was performed with the indicated antibodies. β-tubulin was used as a loading control. d, days. w, weeks. m, months. LNCaP cells were cultured under CSS condition for 3 d, then, were used for the following series of experiments (C-H). **C** Protein levels and subcellular distribution of PHB1 was determined by Western blot and subcellular fractionation. Left: total expression; Middle: nuclear (N) and cytosol (C) expression; Right: plasma membrane expression. Western blot was performed with the indicated antibodies. PHB1 bands were normalized to β-tubulin bands (total and cytosol expression)/Lamin A/C bands (nuclear expression)/ Na^+^-K^+^-ATPase bands (plasma membrane expression). Representative images are shown. **D** Subcellular distribution of PHB1 and AR was analyzed by IF assay. Representative images are shown with a 5 µm scale-bar. PHB1: green; AR: red; DAPI: blue. Magnified images from the regions marked by rectangles showed in the bottom right panel. Colocalization signal is yellow (black arrows). **E** PHB1-AR interaction in whole cell lysate (left) and nuclear extract (right) was determined by Co-IP assay. Cell lysates were immunoprecipitated with anti-PHB1. IgG serves as negative control. **F** Binding of AR to the ARE enhancer regions of TMPRSS2 (above) and PSA (below) genes were monitored by ChIP assay. Cell lysates were immunoprecipitated with anti-AR. Purified rabbit IgG was used as a negative control. **P < 0.01, ***P < 0.001. **G** Phosphorylation level of c-Raf^Ser338^ and MEK with transfection of siNC/siPHB1 was determined by Western blot. Western blot was performed with the indicated antibodies. β-tubulin was used as a loading control. Densitometry analysis was performed using ImageJ, with target protein bands normalized to β-tubulin bands. Representative images are shown. **H** Subcellular distribution of PHB1 and mitochondria were analyzed by IF assay. Representative images are shown with a 5 µm scale-bar. PHB1: green; MitoTracker: red; DAPI: blue. Magnified images from the regions marked by rectangles showed in the upper/bottom right panel. Colocalization signal is yellow (black arrows). *FBS* Fetal bovine serum. *CSS* Charcoal-stripped serum. *d* Days

AR signaling plays a vital role in PCa progression [2]. To elucidate the underlying mechanism by which PHB1 promotes castration resistance, we first determined whether PHB1 is an AR-related protein. Androgen-starved LNCaP cells were stimulated with DHT for indicated times (0\6\12\24\48 h) or concentrations (0\0.01\0.1\1\10 nM). In consistent with Bevan's results [37], Western blot and qRT-PCR results showed that DHT treatment inhibited the expression of PHB1 in a time-/concentration-dependent manner (Fig. 3A), indicating that PHB1 is an androgen suppressive gene. Moreover, the GSE59986 dataset [52] analysis revealed that PHB1 mRNA expression in LNCaP cells was upregulated significantly under chronic androgen depletion. Comparison of PHB1 protein expression levels between LNCaP and LNCaP-AI cells presented a similar trend (Fig. 3B).

Many reports indicated that PHB1 can shuttle between subcellular compartments under the induction of various regulatory factors, and in turn mediate the regulation of several signal pathways [18, 22, 28, 34]. We thereby investigated whether ADT affects the subcellular distribution of PHB1. The ADPC LNCaP cells were cultured under CSS condition for 3d to mimic ADT. As shown in Fig. 3C, Western blot and subcellular fractionation assay showed upregulation of PHB1 expression and changes in its subcellular distribution, that is, nuclear PHB1 decreased but cytoplasmic and cell membrane-associate PHB1 increased significantly compared to FBS group. In PCa cells, the nuclear PHB1 function as a co-repressor of AR, and normal levels of endogenous PHB1 can inhibit androgen-dependent AR activity in LNCaP cells under FBS culture. PHB1 can recruit other co-repressors (Rb, HDAC, BRG1, etc.) and AR to form transcriptional repression complex via indirectly interacting with AR, resulting in inhibition of AR signaling [38, 53]. Immunofluorescence colocalization assay revealed that in cytoplasm, both the PHB1 and AR signals were significantly enhanced, whereas in nucleus, both the PHB1 and AR signals were significantly attenuated. And the colocalization signals of nuclear PHB1-AR almost disappear (Fig. 3D). This observation was confirmed by Co-IP experiments to monitor the PHB1-AR interaction both in whole cell lysate and nuclear extract (Fig. 3E). Furthermore, ChIP results exhibited little binding of AR to the androgen response element (ARE) enhancer regions of two essential AR target genes, TMPRSS2 and PSA, compared to FBS group (Fig. 3F).

The plasma membrane-located PHB1 directly interacts with C-Raf, which is indispensable for the phosphorylation at S338 of C-Raf and its full activation mediated by KRAS [20, 21]. Since CSS 3 d induced the plasma membrane translocation of PHB1, we next determine the activation status of C-Raf/MEK. As shown in Fig. 3G, the abundance of P–C-Raf ^ser338^ and P-MEK increased significantly, but this increased effect was partially attenuated by knockdown of PHB1. Unni et al*.* have demonstrated that cytoplasmic AR can form tertiary complexes with MNAR/Src to constitutively activate MEK in a ligand-independent manner, promoting the androgen-independent phenotype transition of LNCaP [54]. Our result suggested that both the membrane-located PHB1 and cytoplasmic AR/MEK axis contribute to the overactivation of MEK induced by ADT.

Cytoplasmic PHB1 is localized predominantly in mitochondria, where it plays a critical role in maintaining normal mitochondrial function and morphology as well as modulating mitochondrial apoptosis pathway [12, 14, 55]. Upregulation of mitochondrial PHB1 prevents apoptosis by maintaining the mitochondrial transmembrane potential, inhibiting cytochrome c release and caspase-3 activation, and enhancing the expression of Bcl-2 and Bcl-xL [55–57]. Finally, immunofluorescence colocalizaion analysis confirmed that CSS 3d also significantly increased PHB1 abundance in mitochondria (Fig. 3H). These data suggest that PHB1 is upregulated and exports from the nucleus in response to ADT signal, which in turn decreases the inhibition of nuclear AR activity as well as activates C-Raf/MAPK pathway and prevents mitochondrial apoptosis, to mediate its effect in promoting castration resistance.

### FL3 has a more potent inhibition effect on ENZ-sensitive CRPC, particularly combined with ENZ

**Fig. 4:**
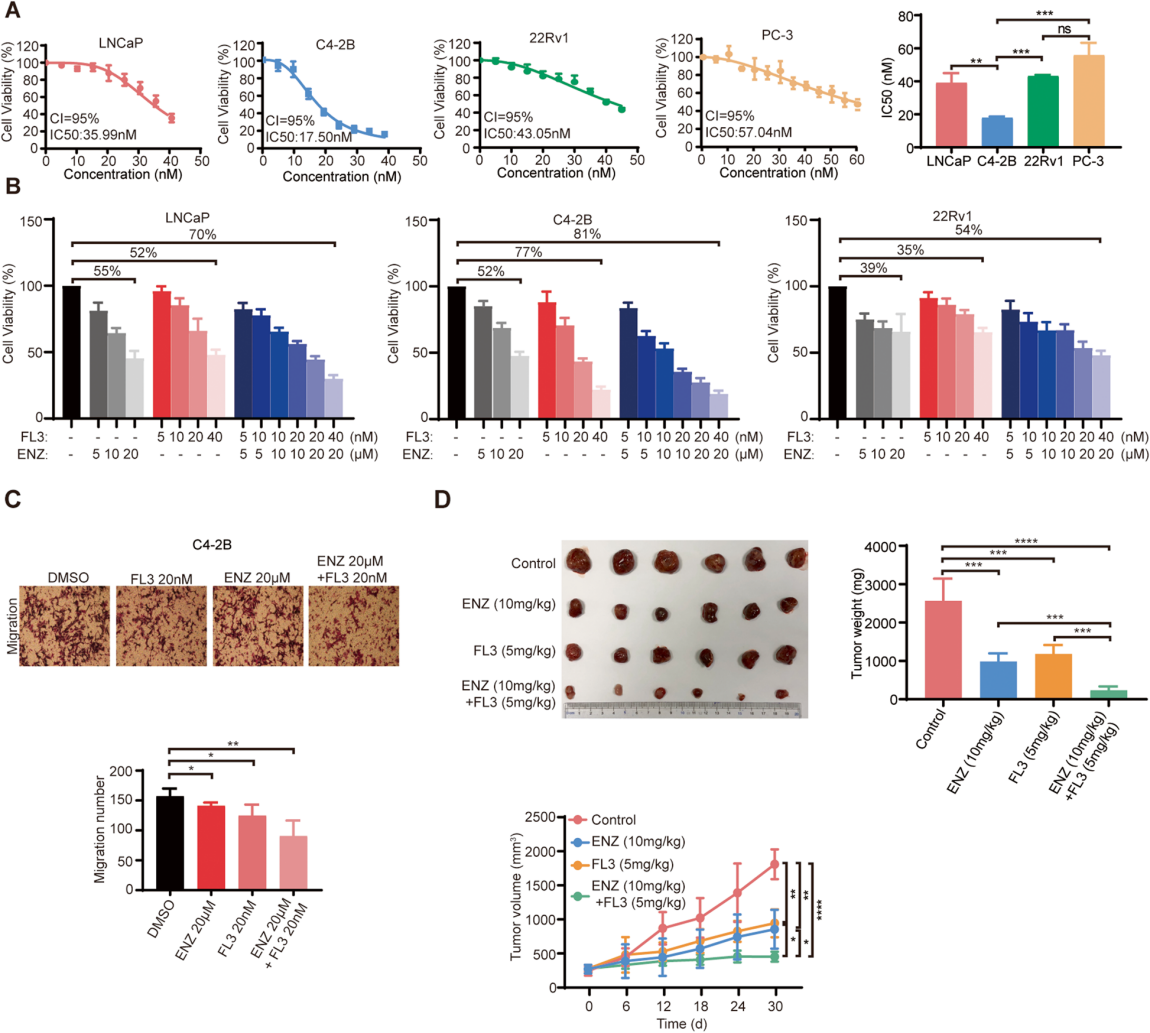
FL3 has a more potent inhibition effect on ENZ-sensitive CRPC, particularly combined with ENZ. **A** IC50 values of FL3 in LNCaP, C4-2B, 22Rv1 and PC-3 cells were determined by MTS assay. The absorbance was read at 490 nM. Representative results and the representative bar diagram of three independent experiments are presented. **P < 0.01, ***P < 0.001. *ns* no significance. **B** Cell viability of LNCaP, C4-2B, and 22Rv1 cells was determined by MTS assay. Cells were treated with ENZ, FL3, and FL3 combined with ENZ in indicated concentrations for 48 h. The absorbance was read at 490 nM. **C** The effects of FL3 alone and/or in combination with ENZ on migration of C4-2B cells were determined by transwell assay. Above: Representative images; Below: quantitative results of triplicate experiments. *P < 0.05, **P < 0.01. **D** The effects of ENZ, FL3, and FL3 plus ENZ on C4-2B cells in vivo were monitored by castrated mice possessing xenografts. C4-2B tumor-bearing castrated mice were treated with ENZ, FL3, and FL3 combined with ENZ in the indicated concentrations for 30d (*n* = 6). Upper left: photographs of C4-2B tumors collected from sacrificed tumor-bearing mice in each group; Upper right: average tumor weight of C4-2B in each group; below: average tumor volume of C4-2B in each group. *P < 0.05, **P < 0.01, ***P < 0.001, ****P < 0.0001. d, days

Since PHB1 has been suggested to play an important role in promoting castration resistance, we next explored the potential of FL3 targeting PHB1 to treat CRPC. Under the continuous selective pressure from ADT/anti-androgens, AR signaling either undergoes further mutation to reactivate (such as AR-V7 without the ligand-binding domain (LBD) or evolves into AR no/low expression, differentiated into AR^**+**^CRPC and AR^**−/lo**^ CRPC subtypes [6, 58, 59]. For this reason, we first chose ADPC cell line LNCaP, AR^+^CRPC cell line C4-2B and 22Rv1, and AR^−^CRPC cell line PC-3 to determine the half-maximal inhibitory concentration (IC50) values of FL3 on them. The results showed that C4-2B cells were mostly sensitive to FL3 (IC50 17.50 nM), followed by LNCaP (IC50 35.99 nM), 22Rv1 (IC50 43.05 nM) and PC-3 (IC50 57.04 nM) cells (Fig. 4A). We further determined the IC50 value of C4-2B cell with PHB1 knockdown, and the result showed nearly twofold increase (IC50 31.59 nM) compared to the control group (IC50 16.56 nM), suggesting that cytotoxicity of FL3 in CRPC is mainly mediated by targeting PHB1 (Fig. S3A). Based on the measured IC50 values, transwell assay results further showed that 20/40/80 nM FL3 inhibited migration and invasion of all 4 tested PCa cell lines in a concentration-dependent manner, and interestingly, suppressive effect of 40 nM FL3 on migratory behavior of PCa was more pronounced in the C4-2B cells (Fig. S3B-E). Given that the C4-2B cell line with the highest sensitivity to FL3 is also extremely sensitive to ENZ [60], we then used MTS assay to compare the effect of FL3, ENZ, or FL3 combined with ENZ on the proliferation of AR^**+**^ PCa cell lines, LNCaP, C4-2B and 22Rv1, respectively. As shown in Fig. 4B, although the low-dose combination of FL3 with ENZ does not effectively inhibit cell proliferation, the combination of 40 nM FL3 with 20 μM ENZ significantly inhibited the growth of LNCaP, C4-2B, and 22Rv1 cells in 70%, 81%, and 54% inhibition rate (P < 0.001), respectively, which were higher than that by treatment with either 20 μM ENZ (55%, 52%, 39% suppression, P < 0.01) or 40 nM FL3 (52%, 77%, 35% suppression, P < 0.01) alone. We also carried out transwell assays to monitor the effect of FL3, alone and/or in combination with ENZ on cell migration behavior of C4-2B cells. Consistent with the MTS results, the number of migrated cells in the combined administration group was further decreased compared with the single drug administration groups (Fig. 4C).

Finally, we examined the impacts of FL3 on tumor growth of C4-2B xenograft in castrated nude mice. Mice were grouped randomly and treated with DMSO (control)/ENZ/FL3/FL3 plus ENZ, respectively. ENZ or FL3 treatment alone reduced both weight (ENZ vs. control: 983.3 ± 213.7 mg vs. 2566.7 ± 578.5 mg; FL3 vs. control: 1183.3 ± 231.7 mg vs. 2566.7 ± 578.5 mg) and volume (ENZ vs. control: 872.2 ± 284.1 mm^3^ vs. 1809 ± 218.8 mm^3^; FL3 vs. control: 944.2 ± 205 mm^3^ vs. 1809 ± 218.8 mm^3^) of the tumor, and the treatment of FL3 combined with ENZ caused stronger inhibition (Weight, 233.3 ± 103.3 mg; Volume, 453.7 ± 73.1 mm^3^) of tumor growth compared with FL3 or ENZ alone (Fig. 4D). Moreover, there were no significant changes in body weights of all group’s mice (Fig. S3F) as well as their vital organ (liver and lung) histology (Fig. S3G). These results indicate that FL3 significantly enhanced the sensitivity of C4-2B cells to ENZ.

### FL3 inhibits ENZ-sensitive CRPC by affecting the subcellular distribution of PHB1

**Fig. 5:**
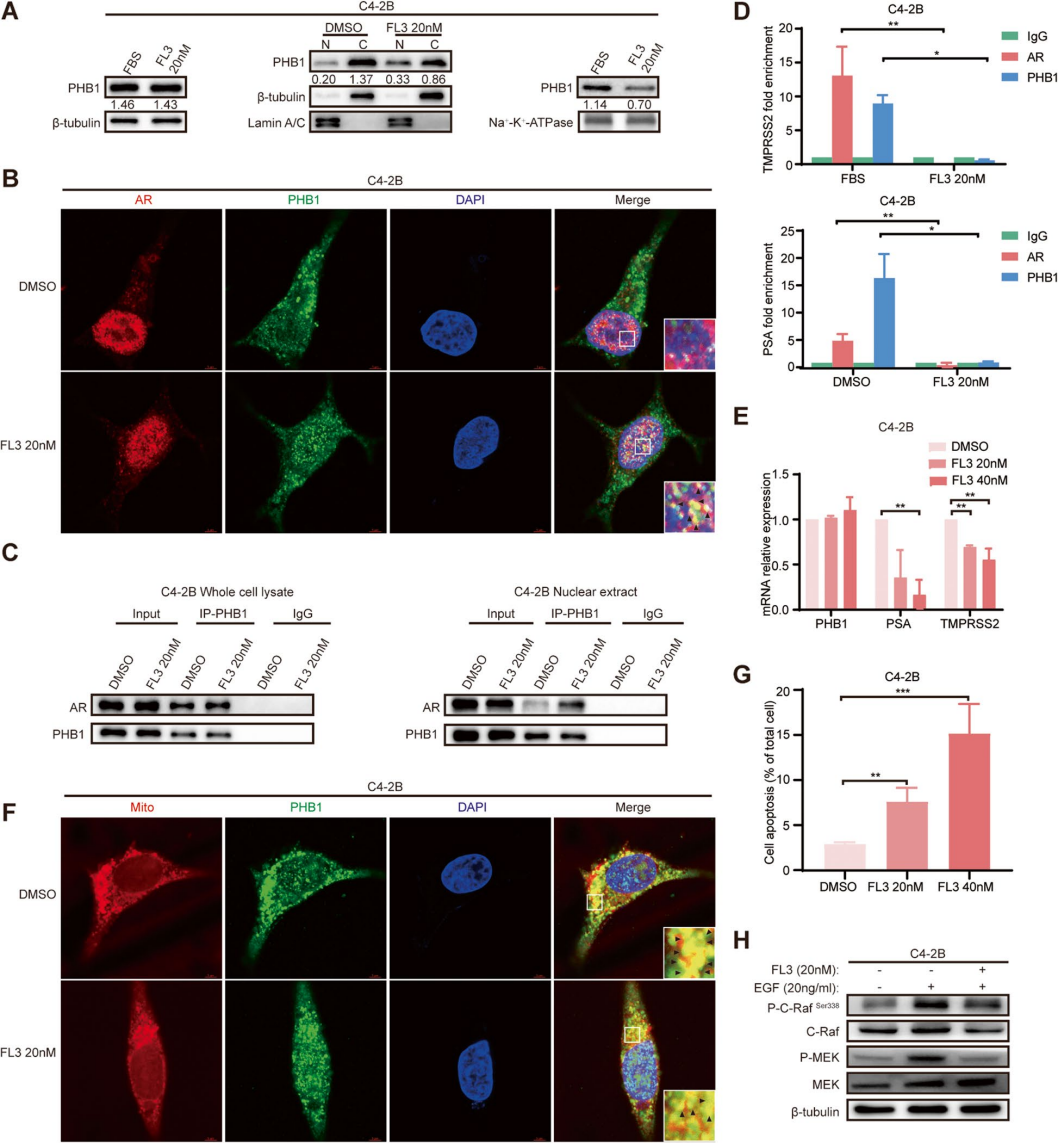
FL3 inhibits ENZ-sensitive CRPC by affecting the subcellular distribution of PHB1. C4-2B cells were treated with DMSO or FL3 20 nM/40 nM for 48 h. **A** The effects of FL3 on PHB1 expression and its subcellular distribution was determined by Western blot and subcellular fractionation. Left: total expression; Middle: nuclear (N) and cytosol (C) expression; Right: plasma membranes expression. Western blot was performed with the indicated antibodies. PHB1 bands were normalized to β-tubulin bands (total and cytosol expression)/Lamin A/C bands (nuclear expression)/ Na^+^-K^+^-ATPase bands (plasma membrane expression). Representative images are shown. **B** Subcellular distribution of PHB1 and AR was analyzed by IF assay. Representative images are shown with a 5 µm scale-bar. PHB1: green; AR: red; DAPI: blue. Magnified images from the regions marked by rectangles showed in the bottom right panel. Colocalization signal is yellow (black arrows). **C** PHB1-AR interaction in whole cell lysate (left) and nuclear extract (right) was determined by Co-IP assay. Cell lysates were immunoprecipitated with anti-PHB1. IgG serves as negative control. **D** Binding of AR/PHB1 to the ARE enhancer regions of TMPRSS2 (above) and PSA (below) genes were monitored by ChIP assay. Cell lysates were immunoprecipitated with anti-AR or anti-PHB1. Purified rabbit IgG was used as a negative control. *P < 0.05, **P < 0.01. **E** mRNA levels of PHB1, AR, PSA, and TMPRSS2 was determined by qRT-PCR. β-actin was used as the reference gene. **P < 0.01. **F** Subcellular distribution of PHB1 and mitochondria were analyzed by IF assay. Representative images are shown with a 5 µm scale-bar. PHB1: green; MitoTracker: red; DAPI: blue. Magnified images from the regions marked by rectangles showed in the bottom right panel. Colocalization signal is yellow (black arrows). **G**. Apoptosis rate of cells was determined by Flow cytometry assay. Representative bar diagram of three independent experiments is presented. **P < 0.01, ***P < 0.001. **H**. The EGF-induced phosphorylation levels of c-Raf^Ser338^ and MEK in cells with siNC/siPHB1 transfection were determined by Western blot. Western blot was performed with the indicated antibodies. β-tubulin was used as a loading control

Previous studies in DLBCL, CRC and UCB suggested that FL3 has different tumor suppressing mechanisms in different tumor types. In different subtypes of the same tumor type, FL3 exhibits different levels of cytotoxicity or even ineffective [23, 33, 34]. To investigate the underlying mechanism by which FL3 inhibits CRPC, we first analyzed the expression and dynamic partitioning of PHB1 upon exposure to 20/40/80 nM FL3 for 48 h in C4-2B, LNCaP, 22Rv1 and PC-3 cells, respectively. The Western blot and subcellular fractionation results showed that 20/40 nM FL3 did not affect total PHB1 protein level but promoted PHB1 translocation from cytoplasm and plasma membrane to the nucleus compared to DMSO control both in C4-2B and LNCaP cells, whereas in 22Rv1 and PC-3 cells, 80 nM FL3 caused significant decreases of the total PHB1 protein without affecting its subcellular distribution (Fig. 5A & Fig. S4A). FL3 treatment did not affect the mRNA level of PHB1 in all 4 tested PCa cell lines (Fig. S4B). We also examined the effect of ENZ on PHB1 expression and its nuclear/cytoplasmic distribution in C4-2B cells and did not observe significant changes in both (Fig. S4C). Furthermore, colocalization analysis using IF assay in C4-2B cells revealed that FL3 promoted the nuclear translocation of PHB1 and consequently enhanced the colocalization of PHB1-AR in nucleus significantly (Fig. 5B), which was further confirmed by Co-IP experiments both in whole cell lysate and nuclear extract (Fig. 5C). Moreover, ChIP results showed that neither AR nor PHB1 was recruited to the ARE enhancer regions of TMPRSS2 and PSA genes with FL3 treatment (Fig. 5D). Previous studies have identified four well-conserved functional AREs in the AR coding region and demonstrated that AR signaling can regulate its own gene expression [61, 62]. We therefore further monitored the expression of AR, PSA, and TMPRSS2 both in C4-2B and LNCaP cells. As expected, the mRNA level of AR, PSA, and TMPRSS2 was significantly downregulated with FL3 treatment, and AR protein level presented similar trend (Fig. 5E & Fig. S4D), suggesting that FL3 inhibited AR transcriptional activity by preventing AR from binding DNA via promoting the PHB1-AR interaction.

Because the nuclear import of PHB1 reduces the cytoplasmic and plasma membrane-located PHB1 in C4-2B cells (Fig. 5A), we further investigated the effect of FL3 in regulating mitochondrial apoptosis and C-Raf/MAPK signaling. As shown in Fig. 5F, 5G, and Fig. S4E, FL3 significantly decreased PHB1 − mitochondria contacts by colocalization analysis along with increased apoptosis rate in C4-2B cells. Meanwhile, FL3 significantly inhibited the EGF-induced P–C-Raf^Ser338^ and P-MEK levels in C4-2B cells (Fig. 5H), suggesting that FL3 also inhibits C4-2B cells in an AR-independent manner. These data strongly support that FL3 exerts its anti-cancer effect via reversing the subcellular distribution alteration of PHB1 induced by ADT in ENZ-sensitive CRPC.

## Discussion

It has been widely reported that PHB1 plays a pro-cancer role in a variety of tumors [8, 63, 64], but there are few reports on the function of PHB1 in CRPC. In this study, data from clinical specimens showed that PHB1 was highly expressed in CRPC and was positively correlated with poor prognosis. Further, gain/loss-of-function analysis showed that under CSS condition, PHB1 can promote the proliferation, invasion and metastasis of PCa cell lines, LNCaP, C4-2B, 22Rv1 and PC-3, respectively. These results strongly support that PHB1 is one of the potential driving factors for castration resistance. In addition, Bevan et al. reported that PHB1 overexpression from stably integrated tetracycline-inducible vectors could repress androgen-stimulated growth of LNCaP cells both in vitro and in vivo [19, 38]. However, our results showed that transient overexpression of PHB1 under FBS condition in LNCaP cells promoted cell proliferation. The difference in experimental methods may be one of the factors that led to the contrary results between Bevan’s and ours.

PHB1 has been suggested to play a dual role of pro-cancer/anti-cancer, depending on its dynamic distribution characteristics in different tumor types [19, 38, 64]. In tumor cells, both plasma membrane and mitochondria-located PHB1 play a cancer-promoting role via overactivation of C-Raf/MAPK pathway and anti-apoptosis, while the nuclear PHB1 mainly functions as a tumor suppressor [64]. PHB1, together with Rb, repress E2F via recruiting co-repressors of HDAC1, NCoR and BRG1/Brm, thus condensing chromatin and silencing gene activation [15–17, 65]. PHB1 also functions as co-activator of p53 [18]. More importantly, Bevan firstly identified PHB1 as a co-repressor of AR in PCa cells [19, 38]. Despite the upregulation of PHB1 in a variety of tumors, its signal-dependent dynamic partitioning between subcellular compartments contributes to its dynamic transition between anti-cancer role and pro-cancer role [29, 64]. ADT is the direct cause for castration resistance, and we observed the significant upregulation of PHB1 expression trigged by CSS culture, which may be attributed to the loss of AR transcriptional inhibition on PHB1 gene. More importantly, PHB1 translocated from the nucleus to cytoplasm and plasma membrane in response to androgen ablation signal, leading to nuclear PHB1-AR colocalization almost disappear, along with mitochondrial PHB1 increase and activation of C-Raf/MAPK signaling. Various mechanisms attributable to castration resistance are mainly summarized as the abnormal activation or dysregulation of AR-dependent signaling pathway and AR-independent signaling pathways, including MAPKs, PI3K/AKT, Src, JAK/STAT3, Ca^2+^, apoptosis, etc. [3, 72]. As a hormone (androgen) sensitive disease, reactivation of AR signaling remains predominant in the molecular mechanism of castration resistance. One of the mechanisms of AR pathway reactivation is alteration of AR cofactors, including overexpression of co-activators and/or reduction of co-repressors, suggesting that AR cofactors are potential therapeutic targets in CRPC [19, 66–71]. Thus, ADT-caused loss of nuclear PHB1 should be one of the reasons explained reactivation of AR signaling in CRPC. Moreover, ADT enhances the non-genomic AR signaling, which can over-activate AR-independent signaling pathways such as MAPK pathway via the cross-talk between cytoplasmic AR and key members of these pathways [3, 72]. Rescue experiment revealed that the activation of C-Raf/MEK resulting from CSS culture was attenuated by si-PHB1, suggesting plasma membrane-located PHB1 also participated in the over-activation of MAPK pathway to promote castration resistance. Taken together, our results strongly supported that PHB1 promotes castration resistance both in an AR-dependent and AR-independent manner, which is mediated by ADT trigged expression increase and nucleus-cytoplasmic translocation of PHB1. Therefore, PHB1 is a novel potential therapeutic target for CRPC.

As a novel synthetic ligand of PHB1, FL3 has been shown to suppress the growth of several tumors [23, 33, 34]. However, the anti-cancer effect and underlying mechanism of FL3 in PCa remains unclear. For the first time, we revealed the therapeutic potential of nanomolar FL3 in 4 PCa cell lines, ADPC cell line LNCaP with full-length AR (AR-FL), CRPC cell line C4-2B with AR-FL, CRPC cell line 22Rv1 with AR-V7, and AR^−^CRPC cell line PC-3 [6, 73]. Interestingly, the IC50 value of C4-2B is the highest among 4 tested PCa cell lines. Given that both of 22Rv1 and PC-3 are resistant to ENZ, whereas both LNCaP and C4-2B are sensitive to ENZ [5, 67, 74], we inferred that FL3 combined with ENZ may have a better effect on AR^**+**^ PCa cells, especially on C4-2B with the highest sensitivity to FL3. As expected, the inhibition effect of FL3 combined with ENZ on C4-2B cell were obviously higher than that of FL3 or ENZ alone both in vitro and in vivo.

We first discovered that FL3 did not affect the PHB1 abundance but promoted its cytoplasm-nuclear translocation both in C4-2B and LNCaP cells, whereas in 22Rv1 and PC-3 cells, FL3 decreased the PHB1 protein abundance significantly without altering its subcellular distribution. Thus, the difference of FL3 cytotoxicity in different PCa cell lines may be attributable to their difference in response to FL3 either on PHB1 expression or on its dynamic partitioning. The molecular mechanism of the difference in response to FL3 by different CRPC subtypes needs to be further studied in the future. Bevan et al*.* reported that by promoting the formation of the AR transcriptional repression complex, nuclear PHB1 either competitively inhibits the binding of co-activator SRC1 to AR, or recruits HDACs and chromatin remodeling complexes to cause chromatin condensation, ultimately inhibiting AR binding to DNA [19, 38]. Our results showed that FL3 promoted nuclear AR-PHB1 interaction and blocked the recruitment of AR in the ARE region, resulting in decreased expression of AR, PSA, and TMPRSS2, further supporting Bevan’s reports. In addition, our investigations clearly showed that FL3 increased the apoptosis and suppressed the EGF-stimulated activation of C-Raf/MEK and in C4-2B cells. Therefore, in C4-2B cells with higher PHB1 levels compared to LNCaP, FL3 reversed nucleus-cytoplasmic translocation trigged by ADT and consequently reversed the development of CRPC both in an AR-dependent and independent manner, which could explain why the C4-2B cells exhibited the highest sensitivity to FL3 among 4 tested PCa cell lines. As the second-generation antiandrogen, ENZ binds to the AR LBD with high affinity, and in turn impairs AR nuclear translocation and its DNA binding [62]. Combinatorial AR targeted therapy that simultaneously target multi-site of AR pathway is a ‘rational’ strategy for treating AR-dependent CRPC [72, 74, 75]. Our findings on the molecular mechanism of FL3 further affirmed that FL3 can synergize the antiandrogenic effect of ENZ in the nucleus and enhance the anticancer effect of ENZ in an AR-independent manner. Here, we proposed that FL3 as an adjuvant to ENZ might be a better combination therapy targeting the ENZ-sensitive AR^+^CRPC subtype, significantly reducing side-effects and augment clinical effectiveness of ENZ.

## Conclusions

**Fig. 6:**
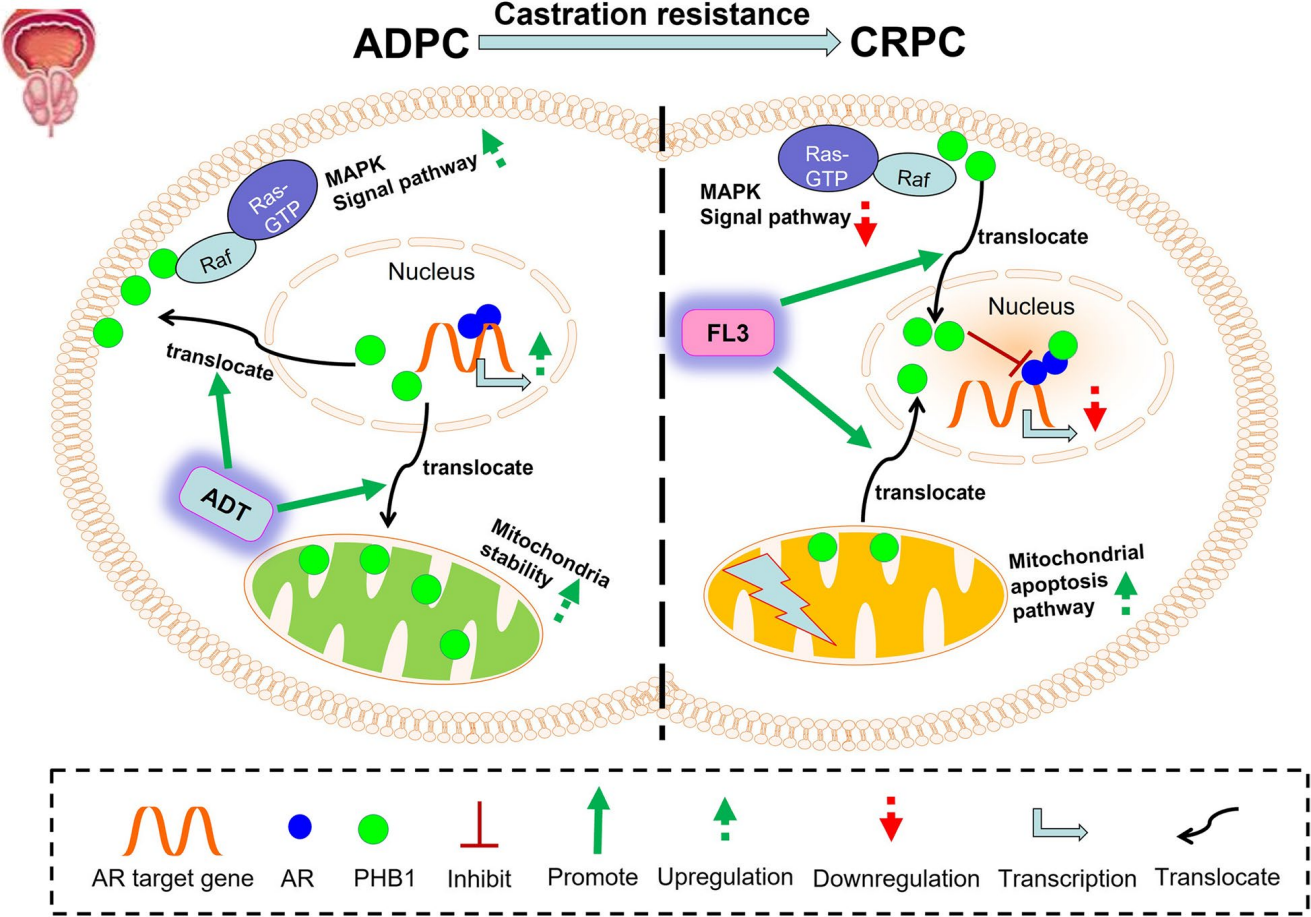
Schematic diagram showing the mechanism by which PHB1 promotes castration resistance and FL3 inhibits CRPC. ADT induces the translocation of PHB1 from nucleus to mitochondria and plasma membrane in ADPC, thereby facilitates reactivation of AR signaling, stabilization of mitochondria and activation of MAPK signaling. While FL3 promotes the translocation of PHB1 from plasma membrane and mitochondria to nucleus in CRPC, thereby inhibits the AR signaling through PHB1-AR interaction as well as MAPK signaling but promotes apoptosis

In summary, our results firstly demonstrated that ADT triggered the anti-/pro-tumor role switch of PHB1 in CRPC by upregulating PHB1 and more importantly, inducing its nucleus-cytoplasmic translocation. While FL3 targeting PHB1 highly inhibits ENZ-sensitive CRPC in AR-dependent/independent manners by reversing the ADT-induced nucleus-cytoplasmic translocation of PHB1. Especially reflowed PHB1 to nucleus synergistically enhance the anti-AR signaling effect of ENZ by forming transcriptional repressive complex with AR and blocking the binding of AR to DNA (Fig. 6), suggesting that combination of FL3 with ENZ to treat ENZ-sensitive CRPC is a promising novel strategy.

## Supplementary Information

**Additional file 1: Table S1. **Primers used in this study.** Table S2. **Antibodies used in this study. **Table S3.** siRNAs used in this study.

**Additional file 2: Supplementary Figures and Legends. Figure S1.** PHB1 expression was significantly increased in PCa tissues and correlated with grades of PCa. **Figure S2.** PHB1 promotes proliferation, invasion, and migration of PCa cells. **Figure S3.** FL3 treatment suppresses the growth, invasion and migration of PCa cells. **Figure S4.** FL3 treatment influences the subcellular distribution or expression of PHB1 in PCa cells and induces apoptosis of C4-2B cells. 

## Data Availability

All data and material during the current study are available from the corresponding author on reasonable request.

## Abbreviations

CRPC: Castration-resistant prostate cancer
PCa: Prostate cancer
PHB1: Prohibitin
AR: Androgen receptor
ADT: Androgen deprivation therapy
ENZ: Enzalutamide
ADPC: Androgen-dependent prostate cancer
DHT: Dihydrotestosterone
CSS: Charcoal-stripped fetal bovine serum
FBS: Fetal bovine serum
TMPRSS2: Transmembrane protease serine 2
PSA: Prostatic-specific antigen
ARE: Androgen-response element
