# Supplementary material for: Targeting PHB1 to inhibit castration-resistant prostate cancer progression in vitro and in vivo
Source: J Exp Clin Cancer Res. 2023 May 20;42:128. doi: 10.1186/s13046-023-02695-0 (PMC10199526; doi:10.1186/s13046-023-02695-0)
Supplement: Supplementary file 1 — Additional file 1: Table S1. Primers used in this study. Table S2. Antibodies used in this study. Table S3. siRNAs used in this study. [file 13046_2023_2695_MOESM1_ESM.docx]

| **Supplementary Table S1. Primers used in this study.** | |  |
| --- | --- | --- |
| **Gene** | **Sense** | **Used for** |
| PHB1 | S:TCATTTTCTCATCCCGTGGGTA | RT-qPCR |
|  | AS: TGCCTGGAGACCAGCTCTCT |  |
| AR | S:GTGGAAGCTGCAAGGTCTTC | RT-qPCR |
|  | AS:TTCAGATTACCAAGTTTCTTCAGC |  |
| PSA | S:AGTCTGCGGCGGTGTTC | RT-qPCR |
|  | AS: TGAGGTCGTGGCTGGAGT |  |
|  | F: GAATCGGGGATCGTACCCAC | ChIP |
|  | R: CTGTGGAAGGGGAGGGAGAG |  |
| TMPRSS2 | S:GCTGCTGGATGACTTGAG | RT-qPCR |
|  | AS: GGCTGCTAAGGCTCTAAGA |  |
|  | F: GAACAGGCCAGGTGAGTGAG | ChIP |
|  | R: AGTAGCTGGGACTAGAGGCG |  |
| β-actin | S:AGTTGCGTTACACCCTTTCTTG | RT-qPCR |
|  | AS: CACCTTCACCGTTCCAGTTTT |  |

| **Supplementary Table S2. Antibodies used in this study.** | |  |
| --- | --- | --- |
| **Antibody** | **Cat no.** | **Used for** |
| PHB1 | cat no. 60092-1-Ig; Proteintech | IHC |
| PHB1 | cat no. sc-377037; Santa Cruz | WB/IP |
| β-tubulin | cat no. AB0012; Abways | WB |
| AR | cat no. 5153; Cell Signaling Technology | WB |
| Lamin A/C | cat no. 4777S; Cell Signaling Technology | WB |
| Na^+^-K-ATPase | cat no. CY5159; Abways | WB |
| P-c-Raf^Ser338^ | cat no. 9427; Cell Signaling Technology | WB |
| c-Raf | cat no. 9422; Cell Signaling Technology | WB |
| P-ERK | cat no. 4370; Cell Signaling Technology | WB |
| MEK | cat no.4604; Cell Signaling Technology | WB |
| PHB1 | cat no. 10787-1-AP; Proteintech | IF |
| AR | cat no. sc-7305; Santa Cruz | IF |
| AR | cat no.5154S; Cell Signaling Technology | ChIP |
| PHB1 | cat no. 10787-1-AP; Proteintech | ChIP |
| Alexa Fluor Plus 488 (green) | cat no. A21206; Invitrogen | IF |
| Alexa Fluor Plus 594 (red) | cat no. A21203; Invitrogen | IF |
| IgG | cat no. sc-2025; Santa Cruz | IP |
| HRP-conjugated Affinipure  Goat Anti-Rabbit IgG (H+L) | cat no. SA00001-2; Proteintech | WB |
| HRP-conjugated Affinipure  Goat Anti-Mouse IgG (H+L) | cat no. SA00001-1; Proteintech | WB |

| **Supplementary Table S3. siRNAs used in this study.** | |
| --- | --- |
| **siRNA** | **Sequence** |
| siNC | TTCTCCGAACGTGTCACGTTTC |
| siPHB1 | S: GCGACGACCUUACAGAGCGUU  AS: UGUCAACAUCACACUGCGCdTdT |
