# Supplementary material for: Targeting PHB1 to inhibit castration-resistant prostate cancer progression in vitro and in vivo
Source: J Exp Clin Cancer Res. 2023 May 20;42:128. doi: 10.1186/s13046-023-02695-0 (PMC10199526; doi:10.1186/s13046-023-02695-0)
Supplement: Supplementary file 2 — Additional file 2: Supplementary Figures and Legends. Figure S1. PHB1 expression was significantly increased in PCa tissues and correlated with grades of PCa. Figure S2. PHB1 promotes proliferation, invasion, and migration of PCa cells. Figure S3. FL3 treatment suppresses the growth, invasion and migration of PCa cells. Figure S4. FL3 treatment influences the subcellular distribution or expression of PHB1 in PCa cells and induces apoptosis of C4-2B cells. [file 13046_2023_2695_MOESM2_ESM.docx]

**Supplementary Figures and Legends**


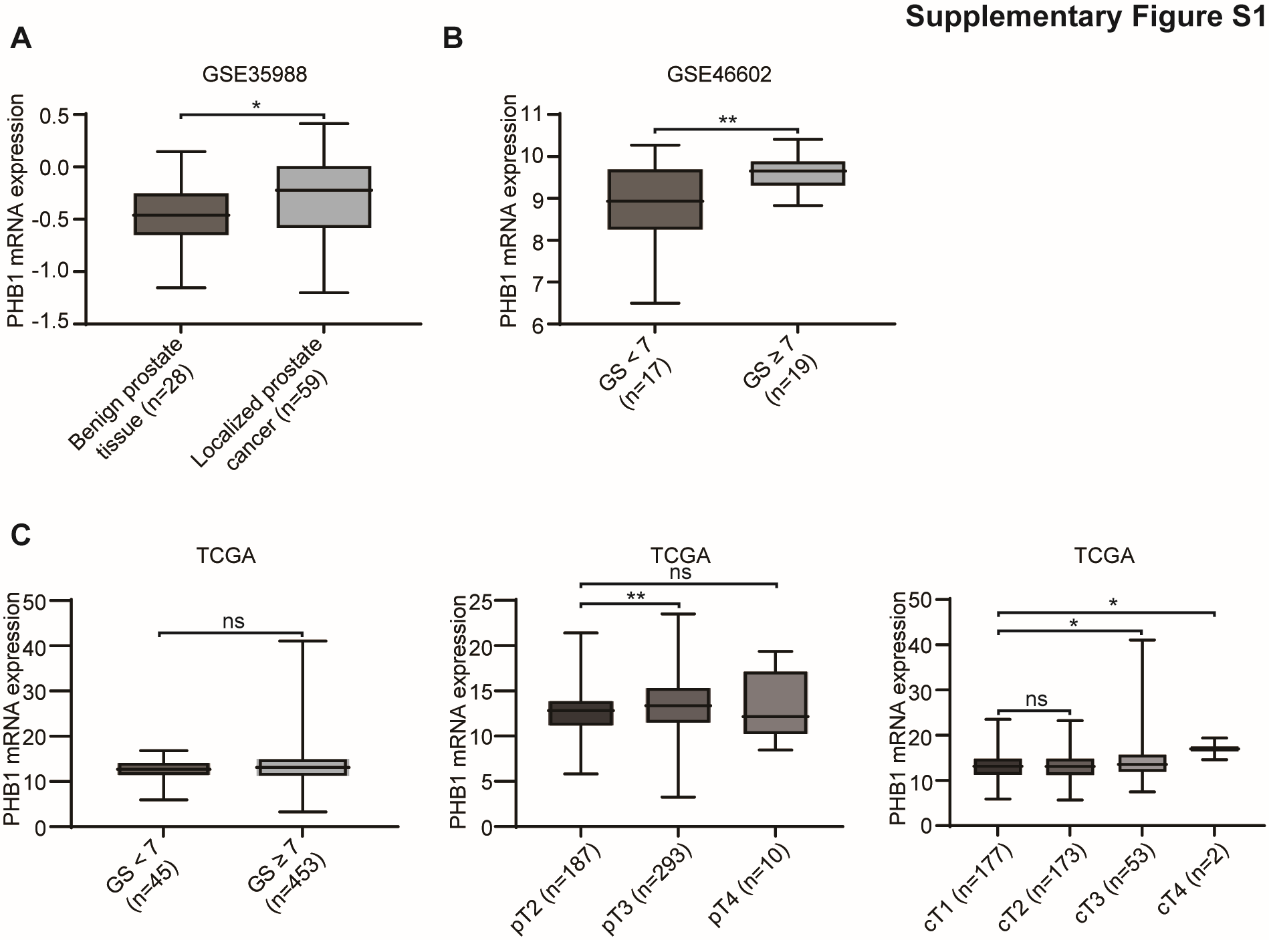


**Supplementary Figure S1. PHB1 expression was significantly increased in PCa tissues and correlated with grades of PCa.**

A. Expression of PHB1 in localized PCa tissues compared with benign prostate samples in public dataset (GSE35988). **P* < 0.05.

B. Expression of PHB1 in PCa cases with Gleason score ≥ 7 compared with those with Gleason score < 7 in public dataset (GSE46602). ***P* < 0.01.

C. Expression of PHB1 in different PCa cases with different Gleason Score (left), pathologic T stages (middle), and Clinical T stages (right) in TCGA database. ns, no significance. **P* < 0.05, ***P* < 0.01.


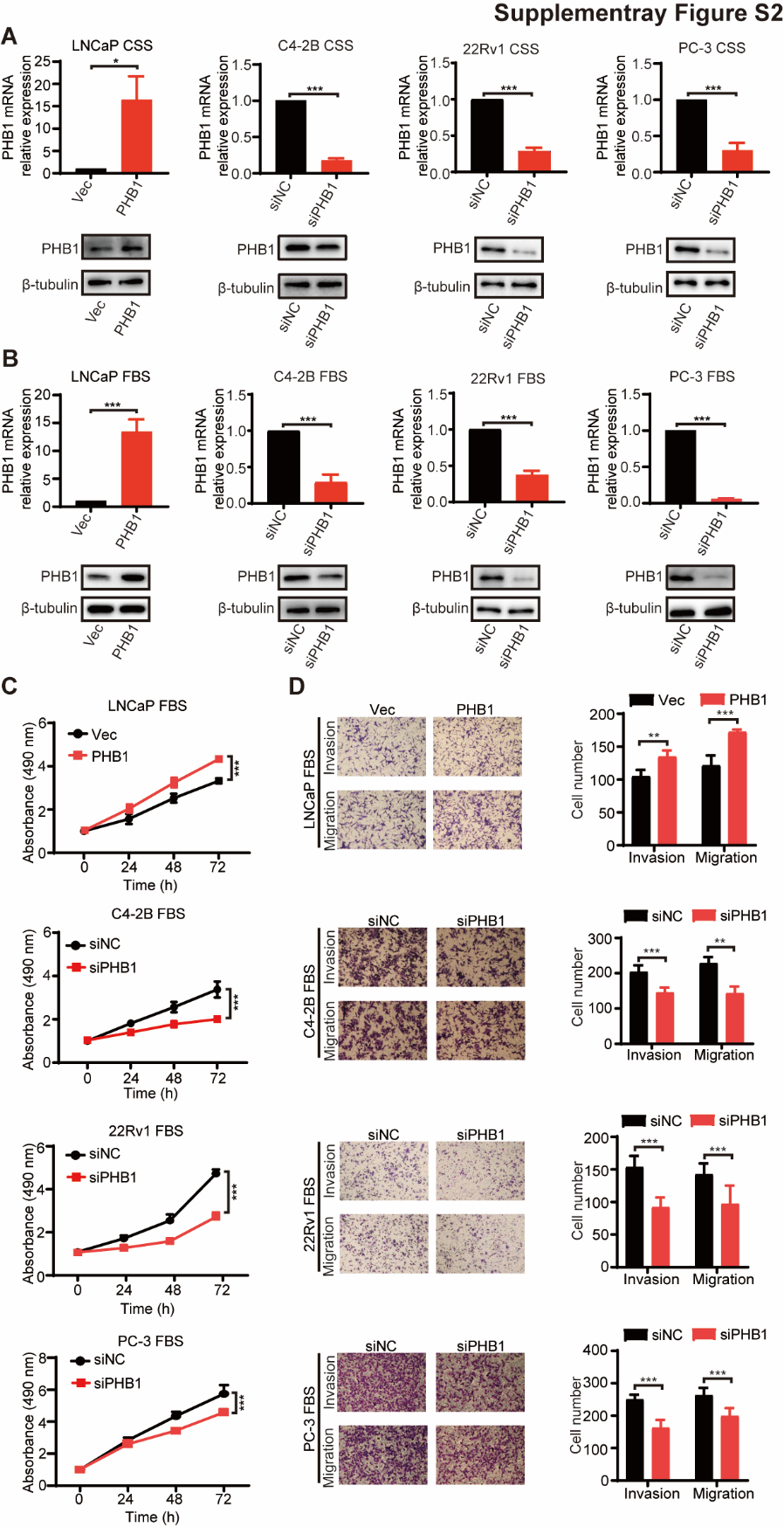


**Supplementary Figure S2. PHB1 promotes proliferation, invasion, and migration of PCa cells.**

The LNCaP cells were transfected with pENTER/pENTER-PHB1, while the C4-2B, 22Rv1 and PC-3 cells were transfected with siNC/siPHB1.

A-B. The mRNA and protein levels of PHB1 were evaluated by qRT-PCR and Western blot under CSS (A)/FBS (B) culture. Western blot was performed with the indicated antibodies. For qRT-PCR, β-actin was used as the reference gene. For Western blot, β-tubulin was used as a loading control. **P* < 0.05, ***P* < 0.01, ****P* < 0.001. CSS, charcoal-stripped serum. FBS, fetal bovine serum.

C-D. Cells were cultured under the FBS medium. Cell proliferation was assessed by MTS assay (C). The absorbance was read at 490  nm at the indicated time points and normalized to 0 h values. Invasion and migration capacities of cells were determined by transwell assay (D). Representative images were shown in the left panel and quantitative analysis is shown in the right panel. **P < 0.01, ****P* < 0.001. FBS, fetal bovine serum. h, hours.


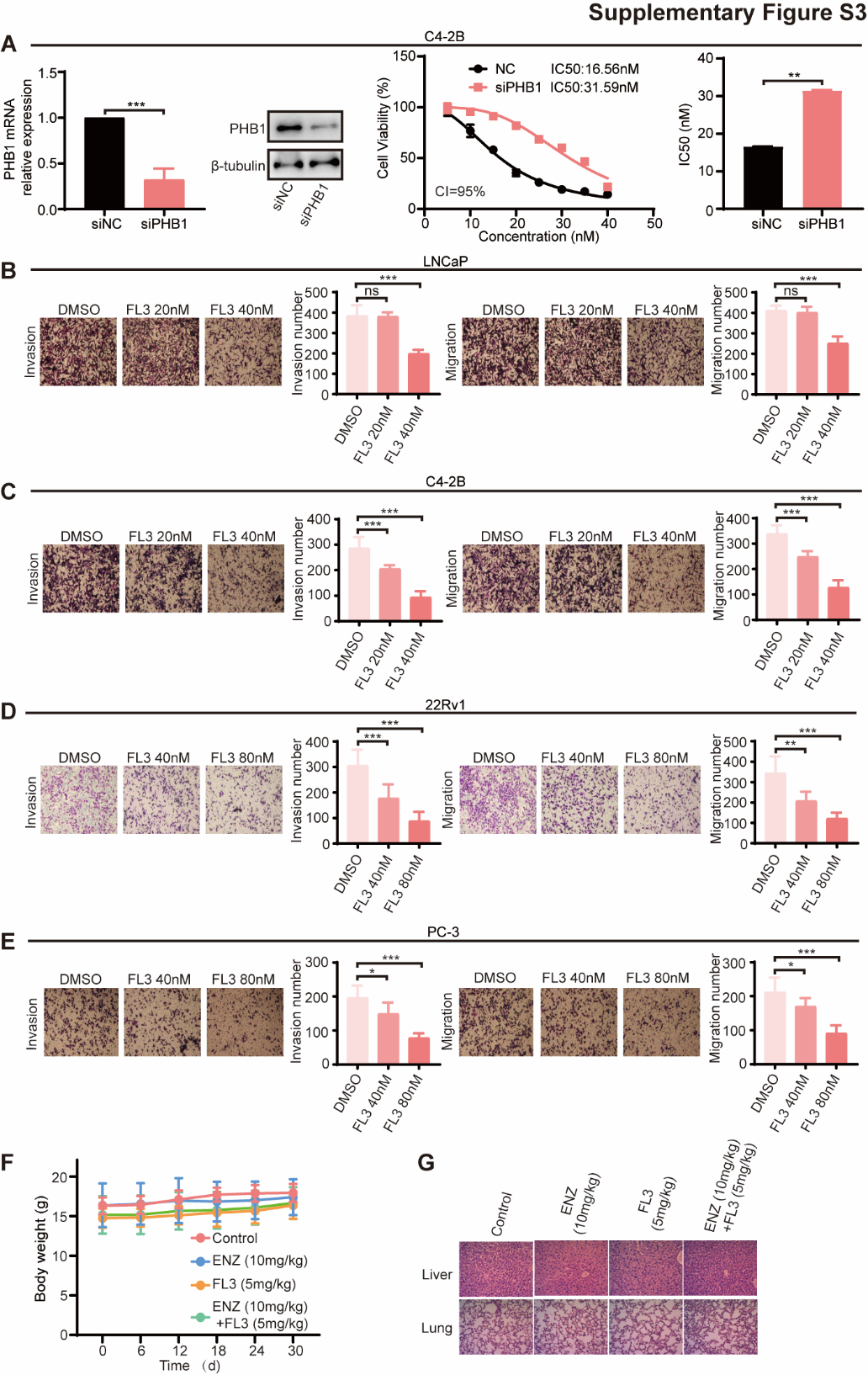


**Supplementary Figure S3. FL3 treatment suppresses the growth, invasion and migration of PCa cells.**

A. The mRNA and protein levels of PHB1 were determined by qRT-PCR and Western blot in siNC/siPHB1 transfected C4-2B cells (left two panels). IC50 values were determined by MTS assay (right two panels). Representative results and quantitative analysis of three independent experiments were presented. The absorbance was read at 490  nm. Western blot was performed with the indicated antibodies. For qRT-PCR, β-actin was used as the reference gene. For Western blot, β-tubulin was used as a loading control. ***P* < 0.01, ****P* < 0.001.

B-E. The effects of FL3 on invasion and migration capacity of cells were determined by transwell assay in PCa cells. LNCaP(B), C4–2B (C), 22Rv1 (D), and PC-3 (E) cells were treated with DMSO or FL3 at the indicated concentrations for 24 h. Invasion (left) and migration (right) of PCa cells to the lower chambers were observed using a light microscope (magnification, ×100); Bar graphs showed the quantitative results of the invasion (left) and migration (right), respectively. **P* < 0.05, ***P* < 0.01, ****P* < 0.001.

F. Average body weight of C4-2B tumor-bearing mice in each group (n = 6). d, days.

G. Drug toxicity in vital organs in each group (n = 6). The livers and lungs were dissected and stained with hematoxylin-eosin. Representative images were shown.


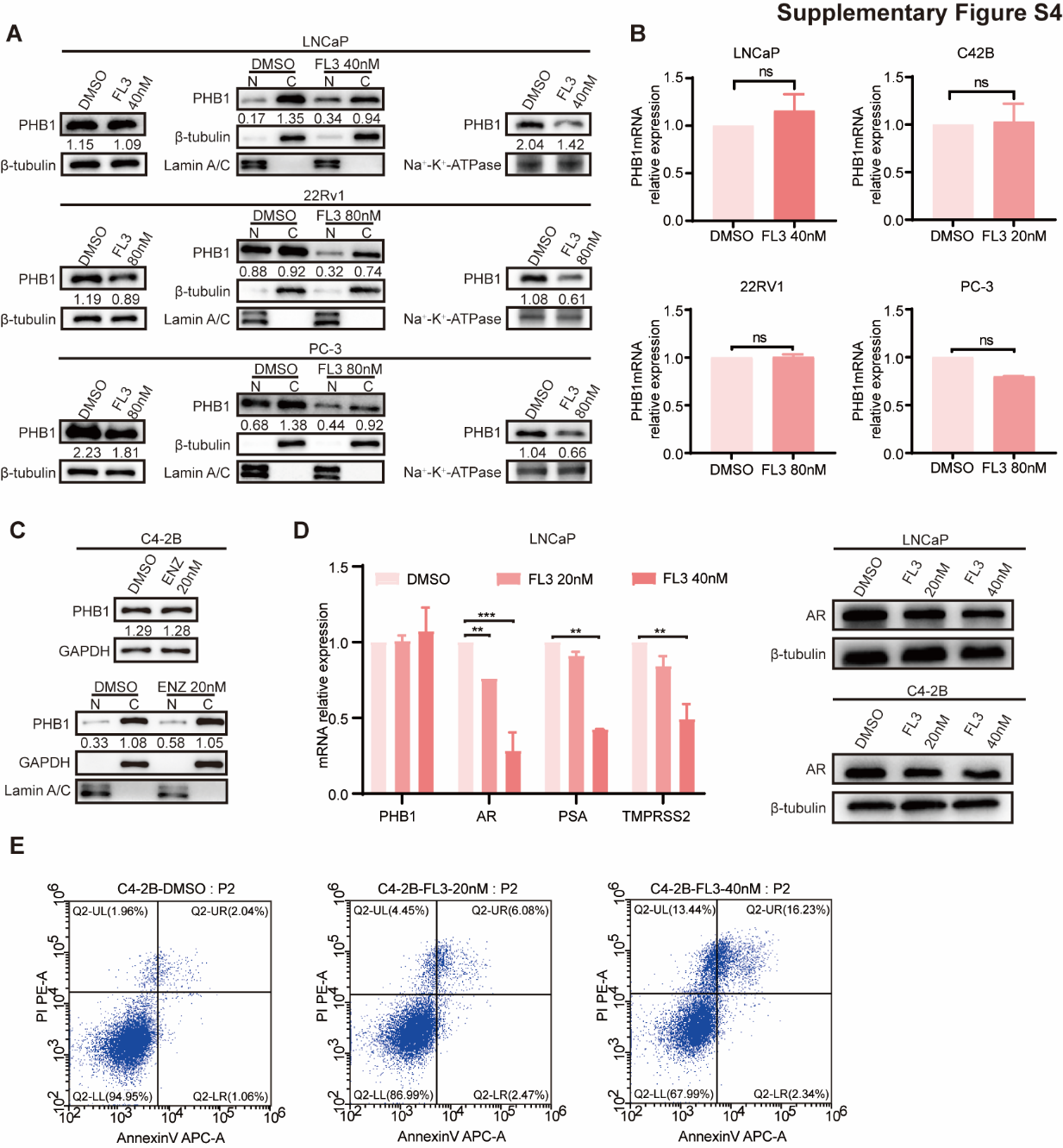


**Supplementary Figure S4. FL3 treatment influences the subcellular distribution or expression of PHB1 in PCa cells and induces apoptosis of C4-2B cells.**

A-B. LNCaP, C4-2B, 22Rv1, and PC-3 cells were treated with DMSO or FL3 20 nM/40 nM/80 nM for 48 h. PHB1 protein expression and its subcellular distribution in LNCaP, 22Rv1, and PC-3 cells were detected by Western blot and subcellular fractionation (A). Left: total expression; Middle: nuclear (N) and cytosol (C) expression; Right: plasma membrane expression. Western blot was performed with the indicated antibodies. Densitometry analysis was performed using ImageJ. PHB1 bands were normalized to β-tubulin bands (total and cytosol expression)/Lamin A/C bands (nuclear expression)/Na^+^-K^+^-ATPase bands (plasma membrane expression). The mRNA expression levels of PHB1 in LNCaP, C4-2B, 22Rv1, and PC-3 cells were detected by qRT-PCR(B). β-actin was used as the reference gene.

C. PHB1 protein expression and its subcellular distribution were detected by Western blot and subcellular fractionation in C4-2B cells. C4-2B cells were treated with DMSO/20 μM ENZ for 48 h. Above: total expression; Below: nuclear (N) and cytosol (C) expression. Western blot was performed with the indicated antibodies. Densitometry analysis was performed using ImageJ. PHB1 bands were normalized to GAPDH bands (total and cytosol expression)/Lamin A/C bands (nuclear expression).

D. The mRNA levels of PHB1, AR, PSA, and TMPRSS2 and the protein levels of AR were detected by qRT-PCR and Western blot in PCa cells with DMSO or 20/40 nM FL3 treatment, respectively. Left: qRT-PCR result of PHB1, AR, PSA, and TMPRSS2 in LNCaP cells; Right: Western blot results of AR expression in LNCaP (above) and C4-2B (below) cells. Western blot was performed with the indicated antibodies. For qRT-PCR, β-actin was used as the reference gene. For Western blot, β-tubulin was used as a loading control. ***P* < 0.01, ****P* < 0.001.

E. Representative images of apoptosis assay on C4-2B cells. C4-2B cells were treated with DMSO or FL3 20 nM/40 nM for 48 h.
